# A Dual Role of Vanadium in Environmental Systems—Beneficial and Detrimental Effects on Terrestrial Plants and Humans

**DOI:** 10.3390/plants10061110

**Published:** 2021-05-31

**Authors:** Ewa Hanus-Fajerska, Alina Wiszniewska, Iwona Kamińska

**Affiliations:** Department of Botany, Physiology and Plant Protection, Faculty of Biotechnology and Horticulture, University of Agriculture in Krakow, Al. 29 Listopada 54, 31-425 Kraków, Poland; alina.wiszniewska@urk.edu.pl (A.W.); i.kaminska@urk.edu.pl (I.K.)

**Keywords:** vanadium bioavailability, human health, soil-plant continuity, pollution of environment, phytoremediation, research progress

## Abstract

The importance of vanadium (V) in the functioning of land systems is extremely diverse, as this element may exert both positive and harmful effects on terrestrial organisms. It recently become considered an element of beneficial character with a range of applications for human welfare. The health-ameliorative properties of this transition element depend on its degree of oxidation and on optimal concentration in the target cells. It was found that a similar relationship applies to vascular plants. However, excessive amounts of vanadium in the environment contaminate the soil and negatively affect the majority of living organisms. A significantly elevated level of V results in the destabilization of plant physiological balance, slowing down the growth of biomass which significantly reduces yield. In turn, low doses of the appropriate vanadium ions can stimulate plant growth and development, exert cytoprotective effects, and effectively enhance the synthesis of some biologically active compounds. We present the scientific achievements of research teams dealing with such topics. The issues discussed concern the role of vanadium in the environment, particular organisms, and highlight its dualistic influence on plants. Achievements in the field of V bioremediation, with the use of appropriately selected microorganisms and plant species, are emphasized.

## 1. Introduction

Many elements that appear in the lithosphere provide nutrients necessary for sustaining living organisms. Vanadium (V) is included as one among elements relatively widespread in the environment. Its concentration in the upper crust of the Earth in some areas may sometimes be similar to that of zinc and nickel. However, due to uneven distribution, the abundance of vanadium in the crust is difficult to estimate. Usually, it is assumed to be approximately 150 mg kg^−1^ (c.a. 0.019% of crust) so V is relatively common in parental rock, soil, groundwater, fossil fuels, and living organisms [1,2,3,4,5]. Volcanic areas with basaltic layers or gabbros are extremely rich in vanadium (c.a. 200–300 mg V per kg of mafic rock) [6,7,8]. The V content in carbonaceous sedimentary rocks was even treated as guideline for petroleum exploration [9,10]. Anthropogenic emissions from fossil fuels, especially as a result of long-term combustion of hard coal or crude oil, contribute to an increase in the level of vanadium to a much greater extent than is the case with any known natural sources [11,12]. Moreover, during coal combustion, elevated levels of vanadium pentoxide (V_2_O_5_) are emitted into atmosphere along with fine particles. The V_2_O_5_ becomes toxic to higher animals, including humans in concentrations exceeding 1 mg L^−1^ [13,14,15,16]. It was ascertained that the concentration of vanadium oxides in an urban environment with a large number of inhabitants can achieve 10 ng per cubic meter of air, while in sparsely populated rural areas is about 1 ng m^−30^, and above the eastern Pacific Ocean the mean vanadium air concentration is as low as 0.1 ng m^−3^ [17,18,19].

This paper provides insights into the results of researches conducted by scientific teams working around the world. Featured results concern both innovative ways of using vanadium in human economic activity and, on the other hand, the environmental threats resulting from the growing demand for the extraction of vanadium-rich ores. Bearing in mind the constantly increasing content of vanadium in the environment, we have undertaken a review of the most important reports on its effects on human health and plant welfare, the latter of which is known to form the basis of the trophic chain. We mainly intended to shed a light on the possible, still insufficiently researched, environmental relationships of this element.

Thus, we were focused on the current research on the interaction of plants and humans with some compounds or ions of this 21st century element, as vanadium is currently termed. In particular, we have noticed a significant gap in the progress of research on the phytoremediation of V-contaminated areas.

## 2. The Economic Use of Vanadium Compounds, the Basic Geochemistry and Elements of Biochemistry of V

In land environments, vanadium compounds are found in over fifty different ores [20,21]. This redox-sensitive metal exists in various minerals in different forms such as, among others, V(II), V(III), V(IV) and V(V), and is found in, for example, vanadates containing sulfides such as patronite, silicates e.g., roscoelite and cavansite or phosphates. In addition naturally occurring vanadium-containing ores include vanadite, carnotite, goethite, and birnessite [22,23,24]. These resources are currently highly exploited, mainly because of the extensive applications of this metal in some important modern industries [25,26,27]. Therefore, continuous mining and smelting activities, especially for vanadium-titanium magnetite, carnotite, vanadinite and other V-containing ores contributes to contamination of vast areas in many countries on most inhabited continents. Canada, Brazil, China, Russia, Australia, South Africa, and some European countries deal with extraction or processing of vanadium compounds [20,26,28,29,30,31], since it is a relevant element in several important industrial fields. The main current applications are the production of high-grade steel metal alloys [32], the building of redox flow batteries (VRFBs) [26], nanomaterials [25], and catalysis [26,33]. In particular, V-catalyzed processes have attracted the attention of scientists [33,34,35,36] because those metal-catalyzed reactions are a powerful tool used to design new sustainable chemical synthetic processes according to Green Chemistry principles [32,37]. Vanadium and zircon are also routinely used in the process of ceramic pigment production [32,33,38].

Therefore, this important transition metal is gradually becoming more widespread in the lithosphere and soil in the form of ores or different types of compounds, respectively. In recent years, increased content of V in soil has been found more and more frequently [10,12,21,39,40]. Aside from iron-bearing minerals, some mafic rock with V content (from 600 to 4100 mg kg^−1^) or rock phosphates (50−2000 mg kg^−1^) are exploited, and bring measurable economic benefits [23,25,26,38]. Thus, it is not surprising that vanadium is found to be present in soils within a range of 10−220 mg kg^−1^, depending on the considered location [3,12,28,31,41,42]. Moreover, it is additionally released to the soil matrix as a result of applying V-bearing fertilizers in agriculture, or in the case of leachates from mine tailings, land-filled municipal sewage sludge, or slag from steel manufacturing [5,30,43]. The presence of vanadium compounds in the soil is currently considered a serious toxicological problem, taking into account the effect that excessive vanadium levels exert on plants and animals.

The biochemical properties of vanadium are also interesting due to the complexity of the chemical behavior of this metallic element, and the great versatility of the coordination sphere around the metal center. Moreover, since V belongs to the transition series, it has a rich redox chemistry and is a typical compound with various degrees of oxidation. From the point of view of biological systems, it is essential that all important valences of this element, i.e., III, IV, and V (that is, from −III to +V) should be examined [44,45,46]. Vanadium(III) and V(IV) compounds are rather unstable in the presence of oxygen at physiological pH. Although V in the oxidation state +III may be found in some minerals, it is virtually absent in environmental solutions because of limited solubility. In turn, despite the fact that vanadium(IV) compounds are easily oxidized to vanadium(V), the stability of the second form is highly dependent on the presence of specific ligands [45]. In biological systems, vanadium(V) is mainly found as vanadate(−V) anions. For organisms, the toxicity of the pentavalent cationic form (+V) is considered six to ten times greater than that of V(+IV) (i.e., vanadyl(+IV)), as it is connected with inhibitions of phosphatases, ATPases, and other important enzymes [47,48,49].

## 3. Vanadium in the Environmental Systems

### 3.1. Vanadium in the Soil and Possibilities of the Use of Microorganisms to Reduce Its Toxicity

**Figure 1:**
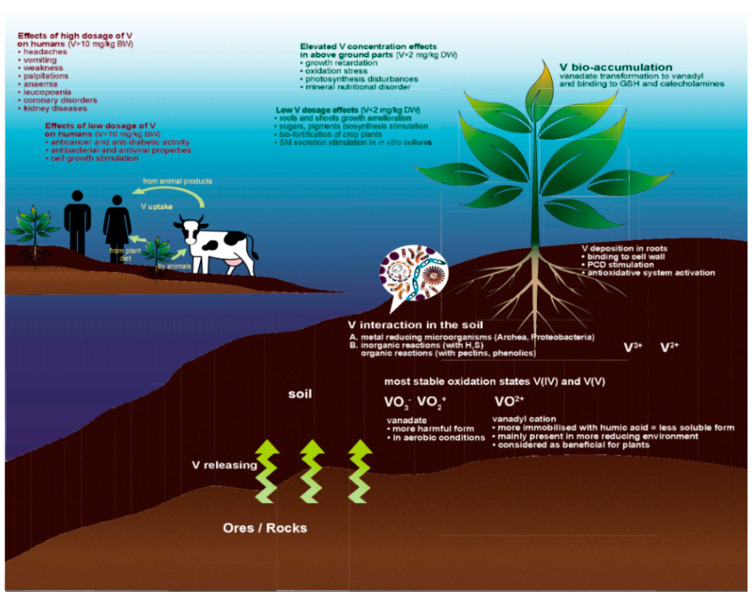
Vanadium circulation in the environment and main beneficial and detrimental effects on plants and humans. (1) V transformation within the soil and roots—marked with white font; (2) V translocation to stems and leaves and its effects on plant physiology—marked with green font; (3) V uptake and its effect on human organism—marked with yellow and brown font; SM—secondary metabolites.

Soil contamination with vanadium is currently an important concern. Due to complex V environmental chemistry, there is no simple solution allowing the limitation of soil pollution and enhancing its cleanup. Vanadium toxicity varies considerably with the nature of the compound under consideration. As explained above, vanadium occur in nature in different oxidation states (mainly from −1 to +5), but the most common are its trivalent, tetravalent, and pentavalent forms [28,49]. However, most published works concern V in the oxidation states V(IV) and V(V) i.e., assessment of vanadyl cations VO^2+^(IV) and vanadate(V) anions, HVO_4_^−,^ and H_2_VO_4_^−^ [28,29,49]. In aerobic conditions, the dominant forms are vanadate(V) compounds, while in reducing environments VO^2+^(IV) is much more frequent. As with other trace elements (TEs), vanadium may be sorbed in or adsorbed on different soil components [31,41,42]. For example, the cation VO^2+^ can be strongly adsorbed on soil particles, and in different pH ranges it may form complexes with humic acids [29]. Therefore, promoting reduction of V(V) forms to lower redox states may be treated as reasonable remediation strategy (Figure 1). The level of soil contamination may be verified, among other ways, on the basis of geoaccumulation index (GI), enrichment factor (EF) or pollution load index (PLI) [50,51,52]. Larson et al. [53] used XANES spectroscopy and HPLC-ICP-MS to study the speciation of vanadium in the soil of a pine forest stand localized in southern Sweden. A field experiment was started there in 1978. At that time, different liming levels were used, i.e., 0.2, 0.7 and 1. kg m^−2^. The authors of the aforementioned paper [53] found that the pentavalent vanadium form was largely accumulated in the mor layer, and they concluded that after 26 years the content of V in studied soil was below toxic the threshold according to Swedish regulations. Using speciation techniques, they determined that the speciation of vanadium in soil depends on properties such as V concentration, particle size distribution, sand, clay and silt content, soil pH, cation exchange capacity (CEC), organic matter content (SOM), and iron and aluminum hydroxide content, as the latter hydroxides have been proven to be important sorbents of V_2_O.

Vanadate is almost a structural and electronic analog of phosphate, especially in the tetrahedral trianionic forms, that is, VO_4_^3−^ and PO_4_^3−^. In most living organisms, vanadate ions negatively influence the phosphate-metabolizing system by inhibiting phosphatases, phosphodiesterases, ribonucleases, ATPases and other enzymes [54,55]. It is therefore important that vanadium(V) may be reduced to a less soluble V(IV) form by inorganic reactions such as with H_2_S [56] or by some metal-reducing microorganisms (Figure 1). The microbiota may reduce vanadium through detoxification or to use this element as an electron acceptor during respiration. A second pathway was found for *Anabena azotica* and *Azotobacter vinelandii* (which have vanadium-dependent nitrogenases for the conversion of dinitrogen to ammonium anions) [52,53]. *Geobacter metallireducens* from the Geobacteriaceae family have been found to be effective vanadium-reducing bacteria [57]. *Shewanella oneidensis* and some strains of *Pseudomonas* have also been reported to be almost comparably effective [58,59,60,61]. Zhang et al. [62] proved that thermophilic methanogen archeons are capable of reducing pentavalent vanadium (vanadate) to vanadyl forms under a variety of conditions. This approach is currently treated as anaerobic vanadium remediation. Thus, both chemical and biological treatments allowing the immobilization of soluble vanadium forms and forming reduced forms should be applied in order to remediate either contaminated ground/waters or the soil water fraction [57,61].

Relatively recently, a sustainable low-cost technology that uses anaerobic microbes that form an electroactive biofilm, the so-called microbial fuel cells (MFCs), was developed. On this basis, very promising instruments for the purification of the fraction of water contaminated with vanadium and other heavy metals (HMs) were constructed [63,64]. The bio-electrochemical device has anode and cathode compartments, which are usually separated by the proton exchange membrane (PEM). In the anode compartment, the appropriate bacterial strains break down different types of contaminated media with organic matter, while generating electrons and protons (which must diffuse through the PEM). After the remediation process, electrochemically reduced metals can be recovered from the cathode surface [65]. In vanadium-MFCs with *Rhodoferax ferriducens* inoculated to the anode chamber, V reduction efficiency reached 67.9% after 240 h. The device could work with a maximum power density of 970.2 mW m^2^ [66,67]. MFCs also have great potential as biosensors for water quality assessment [67].

### 3.2. Vanadium in Soil-Plant Continuity: The Effect of Vanadium on Plants

Plants, to grow and develop, must take up minerals from the soil solution in an ionic form. The uptake of needed elements from the soil by individuals of a given population, and in polluted habitats also of some ballast elements, should be treated as a form of geochemical circulation of elements [68,69,70,71]. Excessive doses of micronutrients or ultra-elements often have a negative or sometimes even lethal effect on plant cells [72,73]. The question of the dose and harmfulness threshold is definitely important here, but in the case of vanadium this parameter has not been established for most species. Long-term exposure to excessive doses of HMs has a negative effect on the processes taking place in individual tissues and organs. Thus, the effect of exposure can ultimately be observed at the level of the whole organism.

As a result of uptake of vanadium ions with further accumulation in the aerial parts, some plant species indicate the current level of V in the soil. The content of V in shoots of indicator species should be directly proportional to the content of soluble forms of this element in soil, and these values should be reflected by a linear function. Also, the concentration in the plant root system increases with leachable vanadium content in soil [74]. Wild-growing species such as *Astragalus confertiflorus, A. lentiginosus, A preussi*, *A. thompsonae, Trifolium pratense* (Fabaceae), *Castillega angustifolia* synon. *C. chromosa* (Orobanchaceae), *Chrysothamnus viscidiflorus*, *Cichorium intybus* var. *foliosum*, *Eupatorium capilifolium* (Asteraceae), and *Allium macropetalum* (Amaryllidaceae) were mentioned as indicator plants for vanadium concentration in soils [75,76,77].

In the majority of cultivated plants, the internal V concentration is about 10 times lower than that in the rhizosphere. When vanadium is already present in plant cells, it tends to be converted to vanadyl forms that are bound either to glutathione, catecholamines, or some low molecular-weight peptides [77,78]. In most tested species, vanadium concentration in the tissues varied with their shoot-root ratio. As for the aboveground organs, generally, leaves (shoots) contain higher vanadium content than fruits and seeds because the plants actively protect the generative progeny from the toxic effects of heavy metals [79].

Somewhat similarly to the case of the use of different species in bio-prospection, it is possible to use plant material to remediate polluted sites [80,81,82]. Examples of species that can be used on vanadium-contaminated surfaces are shown in Table 1. The representatives presented in the table are quite often used in this type of application, such as the most commonly used species from the Brassicaceae family [83,84] as well as from Fabaceae, Chenopodiaceae, Asteraceae [85,86,87,88], and Poaceae [74,89]. As intended, Table 1 contains interesting field-scale studies and shows that successful attempts can be made to biologically clean up V-contaminated soils using higher plants. Laboratory and field experiments have also been conducted to elucidate the effects on plant functioning. The results broaden basic knowledge and facilitate the development of phytoremediation protocols for V-contaminated soils.

Historically, however, vanadium was first identified as a plant growth disruptor. Only in the course of research in the 20th century was it found that V can have not only a negative but also a beneficial effect on plants [90]. There are several factors that influence effect of V on plants, both positive and negative, depending on their layout. Among them, the nutritional status of plants has been mentioned. It was found that higher sensitivity to vanadium pentoxide toxicity was ascertained in plants growing under nutrient stress [90,91]. The detrimental effect of vanadium becomes apparent when the concentration in the tissues exceeds 2 mg·kg^−1^ of dry weight [92]. As in other transition metals, vanadium-exposed plants suffer from growth retardation, resulting from severe nutritional and oxidative disorders. The level of V bioaccumulation in plant organs and the severity of negative symptoms increases with an increase in the V concentration in nutrient medium. A commonly observed response is overproduction of reactive oxygen species (ROS) leading to oxidative stress and cell death [92,93,94,95]. In such cases the efficiency of plant photosynthetic reactions decreases, which can be attributed to the disturbance of chloroplasts, namely the structure of thylakoids and photosystems [93,96,97].

Most plant species exposed to vanadium (in soil or nutrient medium) deposit large amounts of its ions in the roots as compared to the shoots [97,98]. In this case, the tolerance strategy involves restriction of V translocation to the shoot, and stabilization of the element in the root system within the soil-plant continuity. The fine roots gradually turn into organic matter. The ability of the roots to detoxify V can be increased by enlargement of intercellular spaces. Vanadium may bind to cell walls to further limit its mobility in the plant [98]. Tolerant species or ecotypes are able to intensify their antioxidant activity in order to counteract destructive effects of ROS [78,93]. This defense involves enhanced activity of antioxidant enzymes and efficient accumulation of non-enzymatic antioxidants, especially anthocyanins, phytochelatins and glutathione, compared to sensitive genotypes [93,98,99,100]. Research shows that it is possible to select V-tolerant genotypes within one species, as in case of rice [94,100]. Properly selected plant material that exhibits relative tolerance to V, and is able to accumulate it, is crucial for phytoremediation programs. Moreover, the detoxification of V-contaminated substrate can be facilitated by the use of various organic compounds, naturally present in the rhizosphere. Here, caffeic acid and pectins have been found to promote the reduction of vanadium(V) compounds to less toxic forms [101].

**Table 1:** The assessment of the potential of the so far studied species representing various taxonomic origin for use during the reclamation schemes of the polluted substrate or bottom sediments from excessive vanadium content.

Family	Studied Species	The Area under Study	Data on Vanadium Concentration	Phytoremediation Usefulness		Reference
				Phytoextraction	Phytostabilization	
Brassicaceae	*Raphanus sativus*	Field research in Panzhihua city- mining V-Ti magnetite	26.6 mg kg^−1^ d.m. and 12.6 mg kg^−1^ d.m. in leaves and root respectively	may be useful	not surely	[84]
Fabaceae	*Medicago sativa* *Trifolium alexandrinum*	Field research near power station	Leaf V conc. 9.6 mg kg^−1^ d.m. and 13.6 mg kg^−1^ d.m. respectively	not fully not fully	useful fully useful	[87]
	*Glycine max*	Field research in Panzhihua city- mining V-Ti magnetite	Leaf conc. 27.3 mg kg^−1^ d.m.	may be useful	useful	[84]
Chenopodiaceae	*Chenopodium album*	Field research: Shian City, Hubei Province, China	384.3 mg kg^−1^ d.m. in roots r	may be useful	fully useful	[5]
	*Spinacia oleracea*	Field research near power station	Leaf conc. 9.1 mg kg^−1^ d.m.	not fully	not useful	[84]
	*Beta vulgaris*	Field research in Panzhihua city- mining V-Ti magnetite	Conc. in the leaf tissue 6.5–36.3 mg kg^−1^ d.m.	may be useful	not surely	[87]
Lamiaceae	*Mentha* × *piperata*	Field research near power station	Leaf conc. 13.0.5 mg kg^−1^ d.m.	not fully	not surely	[87]
Solanaceae	*Solanum tuberosum*	Field research near power station	Leaf conc. 10.8 mg kg^−1^ d.m.	not useful	not useful	[87]
Asteraceae	*Artemisia vulgaris*	Field research at a brownfield site in New Jersey	11.6 mg kg^−1^ d.m. and 113.0 mg kg^−1^ d.m. in leaves and roots respectively	may be useful	fully useful	[74]
Betulaceae	*Betula populifolia*	Field research at a brownfield site in New Jersey	12.1 mg kg^−1^ d.m. and 280.0 mg kg^−1^ d.m. in leaves and roots respectively	may be useful	fully useful	[74]
Anacardiaceae	*Rhus coppallinum*	Field research at a brownfield site in New Jersey	8.7 mg kg^−1^ d.m. and 118.0 mg kg^−1^ d.m. in leaves and roots respectively	not useful	fully useful	[74]
Poaceae	*Phragmites australis*	Field research at a brownfield site in New Jersey	12.1 mg kg^−1^ d.m. and 280.0 mg kg^−1^ d.m. in leaves and rhizomes respectively	fully useful	fully useful	[74]
	*Settaria viridis*	Field research: Shian City, Hubei Province, China	Up to 156.9 mg kg^−1^ d.m. and 142.4 mg kg^−1^ d.m. in shoots and roots respectively	fully useful	fully useful	[5,89]
Rhizophoraceae	*Cerriopis decandra*	Field research: Swamp among Lothian Island and the Bengal Bay, India	781.8 µg kg^−1^ d.m., 812.2 µg kg^−^ d.m and 1439.61 µg kg^−^ d.m. in leaves, wood and roots respectively	useful	useful	[102]
Fabaceae	*Medicago sativa*	Innovative plant/microbiota combined approach	Up to 500 mg kg ^−1^	useful	useful	[88]

The stimulating effect of V occurs at low concentrations, not exceeding 2 mg·kg^−1^ of dry weight of plant tissue. The effect depends on the degree of oxidation state of the element. The tetravalent forms are considered to be beneficial to plant performance, while pentavalent forms are clearly harmful. Some plants, such as *Ipomoea aquatica*, are able to reduce V(V) to V(IV) in order to protect the tissues [103]. The positive effect of vanadium is reflected in the stimulation of growth and accumulation of biomass, especially in the formation of new leaves and flowers, as well as in the development of root systems [103,104,105]. It is often associated with an altered nutritional status of particular plant organs [106]. At the cellular level, the synthesis of chlorophylls, amino acids, sugars, and other metabolites, particularly non-enzymatic antioxidants, is enhanced. The enzymatic antioxidant system may be also activated, at low doses of vanadium. The benefits that were reported in plant functioning upon exposure to V are summarized in detail in Table 2.

In agricultural and horticultural practice, vanadium salts can be used to manipulate plant secondary metabolism. It has recently been shown that V can facilitate the biofortification of crops with forms of iodine and that this favors the formation of its organic derivatives [106,107]. Vanadium compounds can also be applied to stimulate the production and secretion of secondary metabolites in suspension cultures for pharmaceutical purposes. Vanadium compounds can be further used to reduce the effects of stress and to improve the growth of plants under unfavorable conditions. Recent studies report on the protective effect of vanadium (IV) complexes during the treatment of plants with pro-oxidant H_2_O_2_. *Arabidopsis thaliana* facing oxidative stress performed better if it had been pretreated with oxidovanadium(IV) complexes [108]. Previously, organic V complexes, such as oxidovanad(IV) were found to possess cyto-protective properties when applied on animal and human cells suffering from oxidative stress [109,110]. Additionally, vanadium treatment can be used to reduce the detrimental effects or prevent accumulation of selected toxic trace metals, such as Cu, Hg, and Pb [104,105].

### 3.3. The Effect of Vanadium on Animals and Humans

Vanadium is usually present at ultra-trace or trace concentrations in higher plants and some animals respectively [110,111]. In humans the similarity between vanadate and phosphate accounts for the interplay between vanadate and phosphate-dependent enzymes, thus, vanadate may fulfill a regulatory function in metabolic processes depending on phosphate. However, for a long time, vanadium has not been considered a beneficial element for humans because food that we consume on a daily basis contains extremely low amounts of V (usually less than 1 ng g^−1^). Recently, vanadium has been recognized as an ultra trace element for animals including humans, so called occasionally beneficial element [112]. Parsley, and leafy vegetables such as lettuce and spinach, and spears of asparagus, some cereal products (e.g., rye flour), black pepper, and mushrooms are listed as good sources of vanadium in the diet [112,113]. They are now considered suitable sources of V in a well-balanced human diet.

Inorganic medicinal chemistry that uses the properties of metal ions to design compounds with therapeutic applications is currently a dynamically developing research area. The goal is an innovative treatment of several types of human diseases [47,48,114,115]. Vanadium compounds administered at pharmacological doses have shown interesting biological effects, such as anticancer activity [47,114,116,117,118,119], anti-amoebic activity, and beneficial effects in the treatment of some parasitic diseases [1,120]. These compounds have also been found to possess antibacterial and antiviral properties. There are reports of anti-tuberculosis activity [44,121] and activity against influenza and HIV viruses [44], but unfortunately, a toxic effect of V-therapy given at higher doses has sometimes been also found [122,123]. The risk of adverse health effects of vanadium occurs when V consumption exceeds 10 mg kg^−1^ of body weight (BW) for an adult person [122,124].

Some vanadium derivatives gained the term “growth factor mimetic agent” thanks to the work of Cortiso and Etcheverry’s [123]. Moreover, important results of Cortiso and Etcheverry [123] studies on osteoblast-like URM106 cells clearly show that even though lower doses of vanadate stimulated the growth of cells under in vitro conditions, supplementation with higher doses inhibited cell proliferation. Likewise, the application of vanadyl-stimulated cell proliferation in a dose-responsive manner. Additionally, it has been proven that vanadyl and pervanadate are stronger stimulants of cell growth than vanadate. Vanadium ions affect human cells through signal transduction pathways, including those involved in the action of new vanadium(IV)-trehalose complexes [125], and vanadium(IV) complexes, multiple oxygen donor ligands, such as oxadiacetate (oda) and related derivatives with 2,2′ bypiridine, and 1,10-phenanthroline. Regarding the therapeutic uses of vanadium compounds (VCs), research in medicinal chemistry has focused on improving the oral bioavailability, (bio)distribution, and tolerability of new vanadium therapeutic agents, such as insulin-like vanadium compounds, which have an antidiabetic (insulin-enhancing) effect [126]. Among the surveyed VCs, there are the so called insuline-mimetics or insuline-enhancers, which are usually organic vanadium derivatives, such as *bis*(ethylomalatolato)oxidovanadium(IV) (BEOV) [115,126]. They can be studied in detail in animal models [127], which may speed up studies and accelerate research progress.

In excessive doses V becomes seriously harmful for all living organisms, regardless of whether they are sessile like plants or mobile like animals [111,128]. For humans, vanadium poisoning may be very dangerous and cause dermatitis, green coloration of the tongue, vomiting, headache, weakness, palpitations, anemia, leucopoenia, leukocyte granulation or coronary insufficiency, as well as chronic kidney disease [3,44]. Since considerable vanadium amounts can be supplied to human organism with food cultivated on contaminated land (Figure 1), it has become crucial to monitor the content of this element in the soil and in crops grown for consumption. Moreover, it is very important to elucidate in detail how different plant organisms function in the presence of vanadium, and in particular, the availability and storage of V must be thoroughly investigated at various levels of biological organization.

## 4. Conclusions and Research Prospects

Vanadium is an important transition element. The degree of oxidation, and the concentration and duration of exposure determine the dual role that vanadium can play in living organisms. It is no longer considered just a toxic element, it became an ultra-element that can be widely exploited to improve both human health and plant performance. In nature, vanadium is usually present at ultratrace and trace concentrations in higher plants and animals, respectively. In the field of plant science, many of the presented reports support the dose-dependent effect of vanadium ions on plant physiology. Additionally, awareness of the toxic properties of excessive soil V content is growing, which in turn creates the need for the application of innovative bioremediation methods to prevent soil pollution. Novel data compiled in this review suggest V exploitation as a potent elicitor, a stimulant of secondary metabolite production, and a stress-protective compound that can be used for plant priming towards abiotic stress tolerance. These emerging areas of research broaden our understanding on the conditions of beneficial effects of vanadium on plants, which enables the application of vanadium compounds to facilitate production of agricultural plants. On the other hand, it is necessary to further progress in application research related to the reclamation of polluted environments in accordance with the principles of sustainable development.

While significant progress has been made in the field of vanadium biogeochemistry, the metabolomics of vascular plants capable of detoxifying various types of V ions is currently at a very early stage. To approach this problem, the first challenge would be to identify the appropriate plant material. This is not an easy task because the information required for this identification is scattered across different types of publications. Further knowledge should then focus on the specific effects of vanadium in different models and in experiments conducted at different levels of plant organization. In particular, we need to explain the interrelationships between V and other elements/metabolites in some herbaceous and tree models. In our opinion, the next step should be to standardize the methodology which would favor the integration of data obtained by different research teams. This would be especially important for comparing results obtained from different metabolomics studies.

In addition, another important issue is to address model aquatic organisms. In this particular case, both the correct selection of material and the development of an appropriate methodological approach would allow for an effective diagnosis of ecological threats in various water reservoirs.

The long-term task is to reproduce clones (lines) useful in projects aimed at effective remediation of different vanadium-contaminated environmental compartments.

## Figures and Tables

**Table 2:** Examples of beneficial effects of vanadium on plant physiology.

	V Treatment	Effect	Plant Species	References
Growth amelioration	43–342 µM NH_4_VO_3_ 5–15 µM NH_4_VO_3_ 160–400 µM VOSO_4_·H_2_O 0.32–16.2 µM NaVO_5_	↑ dry biomass of roots, no detrimental effect on leaf and stem biomass ↑ plant height at 5 µM, ↑ no. of flower buds and flower biomass at 5 µM ↑ root volume at 5–10 µM ↑ root biomass at 240 µM ↑ plant height at 0.32 and 0.65 µM ↑ fresh biomass at 0.32 µM	*Ocimum basilicum* *Capsicum annuum* *Phaseolus vulgaris* *Ipomea aquatica*	[104] [90] [96] [103]
Biosynthesis stimulation	5–15 µM NH_4_VO_3_ 8.5–171 µM NH_4_VO_3_ 0.32–16.2 µM NaVO_5_ 3–10 µM NH_4_VO_3_	↑ chlorophyll a, b and a/b ratio in leaf and stem ↑ total free amino acids at 5 µM in leaves and roots, at 15 µM in stems ↑ total soluble sugars in leaves (at all conc.), at 5 and 15 µM in stems ↑ non-protein thiols and glutathione at 8.5–43 µM in leaves and roots ↑ phytochelatins at 8.5–85 µM and 8.5–43 µM in leaves and roots, respectively ↑ chlorophyll a, b and carotene at 0.32–0.65 µM ↑ total phenolic compounds (all conc.) ↑ chlorophyll at 5 µM and carotene at 3 µM	*Capsicum annuum* *Zea mays* *Ipomea aquatica* *Capsicum annuum* *Solanum lycopersicum*	[90] [99] [103] [99]
Enhancement of antioxidant system functioning (enzymatic and non-enzymatic)	128–598 µM NH_4_VO_3_ 427–1709 µM NH_4_VO_3_ 0.63–25.3 µM NaVO_3_·2H_2_O	↑ activity of superoxide dismutase (SOD), peroxidase (POD) and catalase (CAT) (all conc.) ↑ activity of SOD, POD and CAT (all conc.) ↑ activity of SOD, POD, CAT, and ascorbate peroxidase (APX) (all conc.) ↑ ascorbic acid and glutathione (all. conc.)	*Oryza sativa* *Cicer arietinum* *Nicotiana tabacum*	[94] [93] [91]
Improvement of nutritional status	2.8–1094 µM V in soil solution 5–15 µM NH_4_VO_3_	↑ P, Fe, Cu, Zn and Mo in shoots ↑ P, Ca, Mg, Fe, Cu, Zn, Mn, B in roots at 15 µM	*Setaria viridis* *Capsicum annuum*	[89] [90]
